# Thigmomorphogenetic responses of an aquatic macrophyte to hydrodynamic stress

**DOI:** 10.3389/fpls.2015.00043

**Published:** 2015-02-05

**Authors:** Jonas Schoelynck, Sara Puijalon, Patrick Meire, Eric Struyf

**Affiliations:** ^1^Ecosystem Management Research Group, Department of Biology, University of Antwerp, Wilrijk, Belgium; ^2^UMR CNRS 5023 Laboratoire d’Ecologie des Hydrosystèmes Naturels et Anthropisés, Université Lyon 1, Villeurbanne, France

**Keywords:** silica, lignin, cellulose, *Egeria densa*, tensile strength, bending strength, Young’s modulus

## Abstract

The response of aquatic plants to abiotic factors is a crucial study topic, because the diversity of aquatic vegetation is strongly related to specific adaptations to a variety of environments. This biodiversity ensures resilience of aquatic communities to new and changing ecological conditions. In running water, hydrodynamic disturbance is one of the key factors in this context. While plant adaptations to resource stress (nutrients, light…) are well documented, adaptations to mechanical stress, particularly flow, are largely unknown. The submerged species *Egeria densa* was used in an experiment to detect whether the presence or absence of hydrodynamic stress causes plant thigmomorphogenetic responses (i) in terms of plant biogenic silica (BSi), cellulose and lignin concentrations, and (ii) in terms of plant strength. Plant silica concentrations, as well as lignin concentrations were significantly higher in presence of hydrodynamic stress. These physiological changes are accompanied by some significant changes in stem biomechanical traits: stem resistance to tensile forces (breaking force and breaking strength) and stiffness were higher for plants exposed to hydrodynamic stress. We conclude that the response of this aquatic plant species to mechanical stress is likely the explaining factor for a higher capacity to tolerate stress through the production of mechanically hardened shoots.

## INTRODUCTION

Aquatic plants can be exposed to important external mechanical forces resulting from pressure exerted by water movement, particularly in flowing ecosystems such as rivers and streams (Puijalon et al., 2011; Puijalon and Bornette, 2013). The consequences for the plants depend on the magnitude of these hydrodynamic forces and on the plants’ capacity to resist to the effect of these forces (Schutten et al., 2005; Puijalon et al., 2011). Shoot breakage occurs when the forces encountered by plants exceed their capacity to resist breakage (i.e., breaking force; Vogel, 2003; Schutten et al., 2005). Plants’ ability to tolerate water movement without suffering mechanical damage relies either on minimizing the hydrodynamic forces or maximizing its resistance to breakage. When growing under permanent flow conditions, aquatic plants may present important thigmomorphogenetic responses (i.e., developmental responses to external mechanical stimulation; Braam, 2005; Telewski, 2006), which can increase the plant capacity to tolerate hydrodynamic forces. These thigmomorphogenetic responses involve many morphological traits (e.g., reduced plant mass and height, reduced leaf sizes, higher biomass allocation to below-ground organs; Doyle, 2001; Strand and Weisner, 2001), sometimes resulting in extremely modified morphologies such as dwarfed individuals (Puijalon and Bornette, 2013). Studies have demonstrated that these thigmomorphogenetic responses have an adaptive value, reducing the hydrodynamic forces encountered by aquatic plants (Puijalon et al., 2005, 2008), but consequences of these responses for resistance to breakage have rarely been assessed. The resistance to breakage of a plant stem depends on its cross-sectional area and its material strength (Denny, 1988; Niklas, 1992; Vogel, 2003), linked to the proportion of strengthening tissues (Ennos, 1997; Read and Stokes, 2006; Telewski, 2006). Both stem cross-sectional area and proportion of strengthening tissues may be affected by thigmomorphogenetic responses (Bociag et al., 2009), but the actual consequences for aquatic plant resistance to breakage still needs to be investigated.

The strengthening tissue in plants consists mainly of cellulose, which is the majority of plant cell wall material. In the intercellular space, lignin can be incorporated to provide additional rigidity and compressive strength to cell walls. It also renders the cell wall hydrophobic and impermeable to water (Turner et al., 2001). Cellulose is a relatively pure compound of glucose units (i.e., polysaccharides). Lignin, however, does not have a unique chemical formula, but is a complex molecule composed of repeating phenylpropane units composed of an aromatic ring with three carbon side-chains. The phenolic groups vary between plants, further confounding the definition of lignin (Palm and Rowland, 1997). Fundamental physiological research on how these components give strength to plants is mainly restricted to (parts of) terrestrial vegetation. During tree growth, for example, cellulose microfibrils give cell walls tensile strength (Sjostrom, 1993), and lignin encasing the cellulose microfibrils imparts rigidity to cell walls (Hu et al., 1999; Genet et al., 2005). For non-woody species, Carpita et al. (2001) state that it is likely that 15% of the *Arabidopsis* genome is dedicated to cell wall biogenesis and modification, which allows the cell to resist the gravitational forces and/or tensile forces associated with the transpirational pull on a column of water. Turner et al. (2001) showed that mutation of *Arabidopsis*, rendering it incapable of secondary cell wall synthesis, causes the cells to collapse. Additionally, cell wall analysis in barley mutants revealed that the maximum bending stress correlated significantly with the cellulose content but not with lignin. Generally, according to Kaufman et al. (1999), the use of lignin diminishes flexibility. The pathways for cellulose and lignin biosynthesis must thus be regulated in a highly coordinated manner, to achieve the proper deposition of these polymers during secondary wall formation. How this co-ordination is achieved remains largely unclear (Turner et al., 2001).

Apart from strength provided by lignin and cellulose, biogenic silica (BSi) can also provide support to the shoot (Kaufman et al., 1999) by giving structural rigidity to the cell wall (Green et al., 1975) at a 10- to 20-fold lower cost (Raven, 1983). Silica is an energetically cheap stiffening material, promoting upright stature and resistance to lodging (flattening by wind or rain). A first relation between silica, cellulose and lignin for aquatic vegetation was shown by Schoelynck et al. (2010). These authors found interspecific relations between silica and cellulose and antagonistic relations between silica and lignin. Relations could be positive or negative, depending on the growth form of the species (submerged versus emerged). Later, Schoelynck et al. (2012) showed that these relations could hold up intraspecifically in the stems of a single submerged aquatic species (*Nuphar lutea* L.): more silica is associated with less cellulose and with more lignin. This study also demonstrated that, in absence of hydrodynamic stress, Si in the biomass of *Egeria densa* Planch. was positively logarithmically related to increasing ambient Si concentrations, until apparent saturation is reached, beyond which biomass Si concentration stabilized. Next it was demonstrated that exposure to hydrodynamic stress could induce higher Si concentrations in stem and leaf tissue of *E. densa* and *Limnophila heterophylla* (Roxb.) Benth. under experimental conditions and in the tissue of *N. lutea* under natural conditions. The authors however, demonstrated neither the direct effect of hydrodynamic stress on cellulose and lignin production nor the consequences of the changes in silica, cellulose and lignin induced by hydrodynamic stress on the plants’ resistance to this stress (i.e., the plants’ strength).

In this study, we experimentally tested whether Si is involved in plant thigmomorphogenetic adaptations to mechanical perturbation, resulting in higher mechanical resistance. It is hypothesized that in case of mechanical perturbation, the plants take up Si, resulting in higher mechanical resistance due to the association of Si with cellulose and lignin.

## MATERIALS AND METHODS

### EXPERIMENTAL SETUP

Two aquaria (110 L) were filled with tap water (nutrient concentration: 3.65 ± 0.25 mg L^–1^ NO_3_^–^ and 0.15 ± 0.04 mg L^–1^ PO_4_^3–^; pH: 8.4). Dissolved silicon concentration was set at 5.0 ± 0.4 mg Si L^–1^ by adding silicic acid (SiO_2_^*^xH_2_O; Merck, Darmstadt, Germany, DAB certificated). This concentration is on the lower end of what is commonly found in lowland rivers and streams (Struyf et al., 2010). Silica concentrations were carefully monitored by analysis on an ICP-OES spectrometer (iCAP 6000 series, Thermo Scientific, Cambridge, UK) to maintain equal concentrations in both aquaria, with SiO_2_ added when necessary. Nutrient availability was regularly monitored by analysis on a colorimetric segmented flow analyzer (SAN^++^, Skalar, Breda, Netherlands) but did not change significantly during the course of the experiment. In the first aquarium, hydrodynamic stress was created by installing a rotor (Turbelle Stream, Tunze, Penzberg, Germany) in the top one-third of the water column. This rotor created a highly turbulent flow regime with a top speed of 0.5 ± 0.1 m s^–1^ around the plants. This pushed the plants mainly in the downstream direction, but because of the turbulence, they were moving constantly (as in a natural situation). The water was also aerated with a regular air stone. In the second aquarium, the water was almost static as a regular air stone created only little water movement, minimizing boundary layers around the shoots. In each aquarium, 100 mature shoots of the submerged macrophyte *Egeria densa* Planch. (non-commelinoid monocots) were added in 10 groups of 10 individuals each. An extra control group of 10 shoots was kept apart to determine original stem morphological traits (length, diameter and fresh mass) before the experiment. The plants were bought at a plant nursery shop where they were grown in tap water under optimal conditions. The experiment ran for 21 days in a climate chamber at a constant temperature (20°C) and a day–night regime of 14 h light (PAR: 50 μmol m^–2^ s^–1^).

### MEASUREMENTS OF BIOMECHANICAL TRAITS

All plants were entirely defoliated immediately after the experiment. 30 stems were randomly picked per aquarium (three per group), and total length, diameter, and fresh mass of the stems was determined. The diameter was measured with a digital caliper (±0.01 mm) at three different points along the 5 cm basal stem part and averaged per sample. The differences between these data and that of 10 original control stems give an indication of the growth and performance of the plants over the course of the experiment (21 days). Next, the biomechanical properties of this subset of stems from the experiment were measured through (i) bending and (ii) tensile tests using a universal testing machine (Instron 5942, Canton, MA, USA).

#### Bending tests

As three-point bending tests could not be performed due to the too high flexibility of the plant stems, the samples were tested as cantilever beams using a one-fixed end bending test (Hamann and Puijalon, 2013). The basal stem samples (5 cm long) were clamped horizontally at their basal end while a force was applied at their midpoint by lowering a probe at a constant rate of 10 mm min^–1^. We calculated the following biomechanical traits related to bending:

– The bending Young’s modulus (*E* in Pa) quantifies the material stiffness and is calculated as the slope of the stress-strain curve in the elastic deformation region.
– The second moment of area (*I* in m^4^) quantifies the distribution of material around the axis of bending, accounting for the effect of the cross-sectional geometry of a structure on its bending stress. As stem cross section was circular, *I* was calculated as *I* = (π*r*^4^)/4, where r is the radius of stem cross section (Niklas, 1992).
– The flexural stiffness (*EI* in N m^2^) quantifies the stiffness of the stem fragment, i.e., the extent to which the segment resists deformation in response to an applied force, and was calculated by multiplying *E* and *I*.

#### Tensile tests

The stem fragments (approximately 8 cm long) were clamped into the jaws of the testing machine and a constant extension rate of 5 mm min^–1^ was applied to the upper jaw until they broke. We calculated the following biomechanical traits at the sample breaking point:

– The breaking force (in N) is defined as the maximum force that the sample can bear without suffering mechanical failure.
– The tensile strength (in N m^–2^) is calculated as the breaking force per cross-sectional area and quantifies the maximum force that the sample can bear corrected by its cross-sectional area.
– The tensile Young’s modulus (*E* in Pa) is defined as the slope of a sample’s stress–strain curve in the elastic deformation region and quantifies the segment stiffness, i.e., the extent to which the segment resists deformation in response to an applied force.

The diameter of all the stem fragments was measured using a digital caliper (±0.02 mm) at three different points along the sample.

### CHEMICAL ANALYSES

After the strength measurements, the subset of basal stem parts was rejoined with their respective upper parts and all entire 200 shoots were dried at 70°C for 48 h. Stems and leaves were kept separated, but individuals were grouped together per original set of ten shoot, resulting in 40 samples (10 leaf samples and 10 stem samples from the aquarium with hydrodynamic stress and similar for the aquarium without stress). This was needed to obtain sufficient dry matter to perform all chemical analyses. Samples were grounded and homogenized with a mill. BSi was then extracted from 25 mg of dry plant material by incubation in a 0.1 M Na_2_CO_3_ mixture at 80°C during 5 h (DeMaster, 1981). The extracted and dissolved silica was analyzed on a colorimetric segmented flow analyser (SAN^++^, Skalar, Breda, The Netherlands). The extraction in 0.1 M Na_2_CO_3_ at 80° has been well established and tested: it is capable of fully dissolving the BSi from plant phytoliths at the solid–solution ratios and extraction time we applied (Saccone et al., 2007). For lignin analysis, basically two major techniques are often used: “Klason lignin” determination for forestry products (Effland, 1977) and “ADF-lignin” for assessing animal forage quality (Van Soest, 1963). The latter is often referred to as more precise and more reproducible (Ryan et al., 1990; Rowland and Roberts, 1994). Moreover, the Van Soest (1963) method also enables to determine α-cellulose separately from hemicellulose, in contrast to the Effland (1977) method. We used the Van Soest (1963) method to analyze the plant samples for α-cellulose and ADF-lignin. In short, between 0.5 and 1 g of dry plant material was treated with cetyl-trimethylammonium-bromide to dissolve and remove proteins. The remaining material was treated with a 72% sulphuric acid. After weighing and drying (105°C), the α-cellulose content is calculated by subtracting the mass before and after the sulphuric acid treatment and dividing this value by the initial material mass. In a third step the remaining material was ignited at 550°C: the ADF lignin content was then calculated by subtracting the mass before and after ignition and dividing it by the initial material mass. We will further refer to ADF-lignin and α-cellulose as lignin and cellulose.

### STATISTICS

Per aquarium, the 30 biomechanical traits analyses on stems and 20 chemical analyses on stems and leaves were averaged and a standard deviation was calculated. We used a Shapiro–Wilk normality test and a Bartlett test of homogeneity of variances on all datasets. Depending whether the assumptions were met or not, we used a *t*-test test or Kruskal–Wallis chi-squared test respectively.

## RESULTS

All plants survived both treatments and performed well (Table 1). There was no significant difference in the length of the mature shoots between the two treatments (*t*_204_ = 0.73, *p* = 0.46, *t*-test) and between the control and treatment plants (*t*_56_ = –1.13, *p* = 0.26, *t*-test control × stress and *t*_70_ = –1.62, *p* = 0.11, *t*-test control × no stress). This implies no length growth (growth rate = 0 cm day^–1^). The experimental stems did increase significantly in diameter as compared to the controls (*t*_13_ = –8.29, *p* < 0.001, *t*-test control × stress and *t*_19_ = –9.67, *p* < 0.001, *t*-test control × no stress), but did not differ significantly between the two treatments (*t*_38_ = –3.38, *p* > 0.05, *t*-test). This implies a diameter increase of 0.05 mm day^–1^. The experimental stems did also increase significantly in fresh mass as compared to the controls (*t*_90_ = –5.87, *p* < 0.001, *t*-test control × stress and *t*_94_ = –8.75, *p* < 0.001, *t*-test control × no stress), but did not differ significantly between the two treatments (*t*_13_ = –8.29, *p* > 0.05, *t*-test). This implies a mass increase of 50 mg day^–1^.

**Table 1:** **Stem morphological traits (length, diameter, and fresh mass)**.

	**Control**	**No stress**	**Stress**
Length (cm)	23.6 ± 4.0^a^	25.0 ± 5.4^a^	24.5 ± 4.2^a^
Diameter (mm)	1.05 ± 0.23^a^	1.94 ± 0.27^b^	1.72 ± 0.16^b^
Fresh mass (g)	2.06 ± 0.59^a^	3.31 ± 1.04^b^	2.90 ± 0.97^b^

Characters in superscript reflect the significant differences between treatments (control, no stress, stress) for each morphological trait, p < 0.05.

### CHEMICAL TRAITS

**FIGURE 1:**
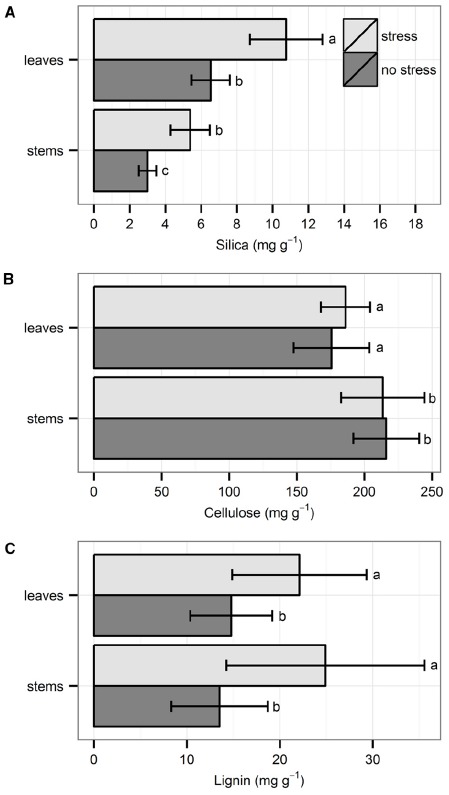
**Chemical properties of the leaves and shoots: (A) silica, (B) cellulose, and (C) lignin.** Data are averages with standard deviation (*n* = 10). Character symbols reflect the significant differences between treatments (stress, no stress) for each plant organ (leaves, stems) and between each plant organ; *p* < 0.05.

Silica concentration was always higher in leaves than in stems. Individuals that were exposed to hydrodynamic stress had significantly higher Si concentrations in both leaves and stems, as compared to individuals not exposed to stress (Figure 1A; χ^2^_1_ = 13.72, *p* < 0.001, Kruskal–Wallis test). Cellulose concentration was always higher in stems than in leaves, but there was no significant difference between the two treatments (Figure 1B; *t*_18_ = 0.20, *p* = 0.84, *t*-test). There was no difference in lignin concentration between stems and leaves, but the concentration was always significantly higher under stressful conditions in both organs (Figure 1C; χ^2^_1_ = 8.17, *p* < 0.01, Kruskal–Wallis test).

### BIOMECHANICAL TRAITS

**FIGURE 2:**
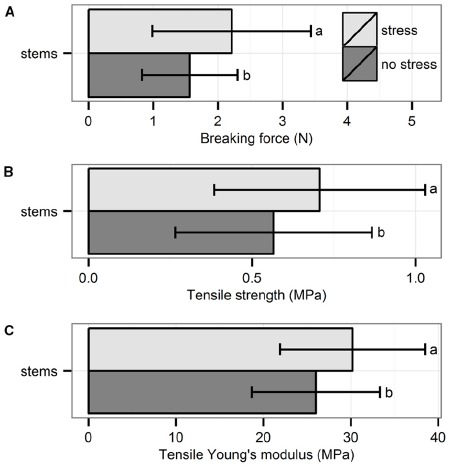
**Biomechanical properties of the stems measured through tensile test: (A) breaking force, (B) tensile strength, and (C) tensile Young’s modulus.** Data are averages with standard deviation (*n* = 30). Character symbols reflect the significant differences between treatments (stress, no stress); *p* < 0.05.

The three biomechanical traits measured through tensile tests were significantly higher for plants exposed to hydrodynamic stress (Figure 2): breaking force (χ^2^_1_ = 4.76, *p* = 0.03, Kruskal–Wallis test), tensile strength (χ^2^_1_ = 4.26, *p* = 0.04, Kruskal–Wallis test) and tensile Young’s modulus (χ^2^_1_ = 4.28, *p* = 0.04, Kruskal–Wallis test).

**FIGURE 3:**
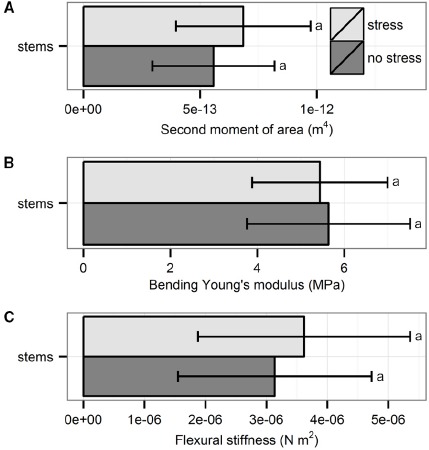
**Biomechanical properties of the stems measured through bending tests: (A) second moment of area, (B) bending Young’s modulus, and (C) flexural stiffness.** Data are averages with standard deviation (*n* = 30). No significant differences were found; *p* > 0.05.

The three biomechanical traits measured through bending tests did not differ significantly between plants from the two treatments (Figure 3): second moment of area (*t*_56_ = 1.76, *p* = 0.08, *t*-test), Young’s modulus (*t*_54_ = –0.44, *p* = 0.66, *t*-test) and flexural stiffness (χ^2^_1_ = 1.07, *p* = 0.30, Kruskal–Wallis test)

## DISCUSSION

In accordance with our hypothesis, the results demonstrate that some biomechanical traits of *Egeria densa* were significantly affected by mechanical stimulation: exposure to hydrodynamic stress led to higher resistance to tensile forces (higher breaking force and tensile strength) and higher stiffness in tension, but did not modify the biomechanical traits in bending. Due to their high flexibility, the shoots of submerged aquatic plants exposed to flow generally align in the flow direction, and are consequently mostly subjected to tensile forces induced by hydrodynamic forces (Usherwood et al., 1997; Schutten and Davy, 2000; Puijalon et al., 2011). A high breaking force and tensile strength represents thus an important adaptation to flowing waters, reducing the risk for the plants to suffer mechanical failure of their shoots (Usherwood et al., 1997; Puijalon et al., 2011; Miler et al., 2012). The thigmomorphogenetic response to hydrodynamic stress observed in the present study may thus have an important adaptive value in increasing the capacity of the plants to tolerate the stress encountered through the production of a mechanically hardened phenotype. The response time is also rather fast as the experiment lasted for only 21 days. The present study demonstrates that the aquatic plants can adapt relatively quick to longer periods of hydrodynamic stress (order of weeks) not only through the production of a phenotype reducing the hydrodynamic forces (Puijalon et al., 2005, 2008), but also through the production of phenotypes increasing the mechanical resistance (i.e., higher resistance to breakage). Adaptation to mechanical stress through production of morphologies mechanically more resistant has been demonstrated for a long time on terrestrial plant species exposed to wind (e.g., Jaffe et al., 1984; Telewski and Jaffe, 1986), on marine algae exposed to waves (e.g., Biedka et al., 1987; Anderson et al., 2006), but not on aquatic plants (Bociag et al., 2009).

This research also confirms the earlier relation between lignin and biogenic Si deposition observed in submerged macrophytes (Schoelynck et al., 2010), and the observed silica uptake by macrophytes in response to hydrodynamical stress (Schoelynck et al., 2012). Emerging research links lignification and biogenic Si metabolism (Ghasemi et al., 2013; Zhang et al., 2013). It has been suggested that lignin can act as a precursor for biogenic Si deposition (Zhang et al., 2013), but the actual physiological mechanisms are currently not known. Although research is also still limited, more evidence points towards silica deposition being closely related to cellulose biosynthesis too. It was found that silica is present in beech leaves as precipitates in the walls of the epidermal and parenchymatous cells, either in the middle lamellae or in the cells against the walls or in the cell intersections (Watteau and Villemin, 2001). In this case, BSi and cellulose, hemicellulose and pectic substances are closely associated (Watteau and Villemin, 2001). Later, Shobbar et al. (2008) found that plants have evolved a broad protective mechanism linking the health and growth of the secondary cell wall with resistance to abiotic and biotic stresses, and abscisic acid (ABA) is the mediator of the mechanism. In times of stress, ABA induces transcripts that encode for cellulose synthase and, at the same time, these transcripts appear in so-called silica cells. These silica containing cells are equally important for leaf strength as is the cell wall. In a recent paper, Fraysse et al. (2010) support the hypothesis of the existence of individual molecules H_4_SiO_4_ or small polymers (silica gel, SiO_2_·nH_2_O) dispersed in the organic matrix of the cell wall.

The inclusion of Si uptake as a plant functional trait is important to assess links between plant physiology, plant distribution and plant tolerance to environmental changes, but also to understand the role of vegetation on nutrient fluxes through the watershed (Schoelynck et al., 2014b). There is a growing line of evidence that Si uptake in wetland and aquatic macrophytes has important implications for other biogeochemical cycles. In a recent study, Si content of aquatic macrophytes increased decomposition rate of the litter, even under low quality conditions with a low C/N ratio (Schaller and Struyf, 2013). Interestingly, when shredders were present, Si had an adverse impact on the decomposition rate. Si uptake in aquatic macrophytes has further also been linked to concentrations of both macro- and micronutrients (Schaller et al., 2012; Schaller and Struyf, 2013), with, e.g., CNP ratios altered by Si deposition and reduced uptake of metals. If plants thus adapt to alterations in hydrological conditions, e.g., induced by more frequent occurrence of peak floods after drainage basin build-up or due to increased occurrence of storm rains, this could alter their impact on other biogeochemical cycles. Aquatic macrophytes are known to create biogeochemical hotspots in their growth patches compared to adjacent non-vegetated areas, with increased organic matter storage and nutrient cycling observed in the patches (Sand-Jensen, 1998; Cotton et al., 2006; Kleeberg et al., 2010; Schoelynck et al., 2014a). Interestingly, the engineering capacity of macrophyte patches to trap and store organic matter is itself to a great extent determined by the rigidity of the stems (Gurnell, 2014).

## CONCLUSION

We conclude that the presence of hydrodynamic stress triggers the uptake of silica in *Egeria densa*, which is linked to changes in the lignin concentration. These responses to mechanical stress are likely the explaining factor for a higher capacity to tolerate this stress through the production of mechanically hardened shoots. These thigmomorphogenetic responses can help to improve the survival chances of the species.

### Conflict of Interest Statement

The authors declare that the research was conducted in the absence of any commercial or financial relationships that could be construed as a potential conflict of interest.
